# Factors Influencing the Implementation of Digital Advance Care Planning: Qualitative Interview Study

**DOI:** 10.2196/50217

**Published:** 2024-08-16

**Authors:** Andy Bradshaw, Jacqueline Birtwistle, Catherine J Evans, Katherine E Sleeman, Suzanne Richards, Robbie Foy, Pablo Millares Martin, Paul Carder, Matthew J Allsop, Maureen Twiddy

**Affiliations:** 1 Cicely Saunders Institute Kings College London London United Kingdom; 2 Leeds Institute of Health Sciences University of Leeds Leeds United Kingdom; 3 Sussex Community NHS Foundation Trust Brighton United Kingdom; 4 Whitehall Surgery Leeds United Kingdom; 5 NHS West Yorkshire Integrated Care Board White Rose House Wakefield United Kingdom; 6 Hull York Medical School, Institute of Clinical and Applied Health Research Allam Medical Building University of Hull Hull United Kingdom

**Keywords:** palliative care, electronic palliative care coordination systems, electronic health record systems, advance care planning, end of life care, technology, Normalization Process Theory, NPT, qualitative

## Abstract

**Background:**

Palliative care aims to improve the quality of life for people with life-limiting illnesses. Advance care planning conversations that establish a patient’s wishes and preferences for care are part of a person-centered approach. Internationally, electronic health record systems are digital interventions used to record and share patients’ advance care plans across health care services and settings. They aim to provide tools that support electronic information sharing and care coordination. Within the United Kingdom, Electronic Palliative Care Coordination Systems (EPaCCS) are an example of this. Despite over a decade of policy promoting EPaCCS nationally, there has been limited implementation and consistently low levels of use by health professionals.

**Objective:**

The aim of this study is to explore the factors that influence the implementation of EPaCCS into routine clinical practice across different care services and settings in 2 major regions of England.

**Methods:**

A qualitative interview study design was used, guided by Normalization Process Theory (NPT). NPT explores factors affecting the implementation of complex interventions and consists of 4 primary components (coherence, cognitive participation, collective action, and reflexive monitoring). Health care and social care practitioners were purposively sampled based on their professional role and work setting. Individual web-based semistructured interviews were conducted. Data were analyzed using thematic framework analysis to explore issues which affected the implementation of EPaCCS across different settings at individual, team, organizational, and technical levels.

**Results:**

Participants (N=52) representing a range of professional roles were recruited across 6 care settings (hospice, primary care, care home, hospital, ambulatory, and community). In total, 6 themes were developed which mapped onto the 4 primary components of NPT and represented the multilevel influences affecting implementation. At an individual level, these included (1) EPaCCS providing a clear and distinct way of working and (2) collective contributions and buy-in. At a team and organizational level, these included (3) embedding EPaCCS into everyday practice and (4) championing driving implementation. At a technical level, these included (5) electronic functionality, interoperability, and access. Breakdowns in implementation at different levels led to variations in (6) confidence and trust in EPaCCS in terms of record accuracy and availability of access.

**Conclusions:**

EPaCCS implementation is influenced by individual, organizational, and technical factors. Key challenges include problems with access alongside inconsistent use and engagement across care settings. EPaCCS, in their current format as digital advance care planning systems are not consistently facilitating electronic information sharing and care coordination. A redesign of EPaCCS is likely to be necessary to determine configurations for their optimal implementation across different settings and locations. This includes supporting health care practitioners to document, access, use, and share information across multiple care settings. Lessons learned are relevant to other forms of digital advance care planning approaches being developed internationally.

## Introduction

Palliative care aims to improve the quality of life for people with life-limiting illnesses through a person-centered, multidisciplinary, and holistic approach [1]. The focus on person-centeredness is reflected in health policy both within the United Kingdom and internationally [2-5]. Key to facilitating person-centered care in palliative services is the concept of advance care planning. This involves having planned conversations with a patient around their individual goals, wishes, and preferences for their current and future care [6]. If a person’s preferences are documented and shared, there is evidence of beneficial outcomes. Advance care planning has been associated with better quality of care, helping people to be cared for and die in their usual place of residence, and preventing unplanned hospital admissions [7-9]. However, other studies indicate that it has no impact on patient outcomes or quality of life [10-12]. Despite this uncertainty, advance care planning has been adopted by health care systems internationally as a key feature of person-centered care.

While there are benefits associated with advance care planning, delivery of palliative care requires the involvement of, and communication between, multiprofessional services across different settings of care (ie, hospice, general practice, community-based care, out-of-hours services, hospitals, emergency services, care homes, and social care) [13,14]. To overcome the need for information sharing across multiple health care providers and settings, electronic health record systems are increasingly being used to document and share advance care planning information. This approach has been reported across countries that include the United States [15,16], Australia [17], and the United Kingdom. In the United Kingdom, the use of electronic health record systems for documenting and sharing advance care plans are called Electronic Palliative Care Coordination Systems, often referred to using the acronym EPaCCS.

EPaCCS emerged in response to the Department of Health’s 2008 End of Life Care Strategy, which advocated for improved coordination of care at the end of life for people with life-limiting conditions (ie, cancer and noncancer conditions, including dementia) [18]. Policy drivers for the widespread use of electronic systems (such as EPaCCS) across health and social care providers have continued to the present day [19,20]. This includes, for example, an expectation that care records for all people living with a long-term condition should include a person’s care needs and preferences, and should be shared with all those involved in their care [19]. The development of EPaCCS sought to overcome challenges arising through the fragmentation of health systems that can lead to patients not receiving person-centered care at the right time and in the right place [21,22]. This can result in patient needs not being met, unplanned and avoidable hospital admissions, and patients not being cared for, or dying in a place of their choice [23-25].

EPaCCS have been developed across the United Kingdom since 2008 and multiple variants have arisen. These include standalone web-based electronic registers such as Coordinate my Care which was implemented in London [26], and template forms integrated into already-existing electronic patient records, such as in Leeds [27] and the Key Information Summary in Scotland [28]. Regardless of the mode used for implementing EPaCCS locally across regions, the Palliative and End of Life Care Information Standard specifies the core content that should be recorded and shared (eg, demographic information, diagnosis, medication, advance care planning information, Do Not Attempt Cardiopulmonary Resuscitation decisions, and preferred places of care and death) [29]. The expectation is that once this information is stored, it should be possible to share across all settings involved in the delivery of palliative and end-of-life care, as well as sharing any updates on the care plan.

However, there is widespread variation with regard to how EPaCCS are implemented within local health care systems (eg, who initiates the creation of a record, which settings of care can access and edit information in EPaCCS records) [14] which has resulted in variable levels of interoperability and access [14,30-33]. In part, this may be a factor influencing the low use rates reported for EPaCCS, with 9%-43% of people with palliative care needs having an EPaCCS record created before death [14,27,34,35]. Alongside low use, there is uncertainty about how EPaCCS are being used in routine practice and limited evidence of their impact, inhibiting the development of an evidence base to guide how their implementation might be optimized [13,14,30,36,37]. The aim of this study was to explore health care professionals’ perspectives on factors that influence the implementation of EPaCCS in routine clinical practice across different care settings in England.

## Methods

### Design

We undertook a qualitative interview study. This approach was selected as we sought to develop new insights and knowledge on a relatively understudied topic area [38]. Our study was informed by an interpretative paradigm [39]. That is, we explored the study aim from the standpoint of ontological relativism (the acceptance of multiple, mind-dependent realities) and epistemological constructionism (an appreciation that knowledge generated during data analysis and write-up reflected interpretations made collectively by the research team) [40]. We reported the research in line with the consolidated criteria for reporting qualitative research (COREQ [consolidated criteria for reporting qualitative research], see Multimedia Appendix 1) [41].

### Theoretical Perspective

EPaCCS can be conceptualized as a complex intervention. This is because they comprise multiple interacting components and operate at the interface of different health care professionals, organizations, settings of care, and patients and their families [42]. Normalization Process Theory (NPT) is an implementation theory that is used to explain how complex interventions are normalized (ie, deeply embedded into, and used as part of, routine practice) [43-45].

In explaining the different types of “work” that people do in normalizing complex interventions, NPT consists of 4 interlinked primary constructs (Table 1) [46,47]. We used these primary constructs of NPT as a guiding theoretical framework to guide data collection, analysis, and interpretation in understanding the factors affecting the implementation of EPaCCS into routine clinical practice.

**Table 1:** An overview of the 4 constructs from the Normalization Process Theory (NPT), definitions of the constructs, and how the constructs were applied and understood in the context of the study.

NPT construct	Definition^a^	Framework applied in this study^b^
Coherence (“sense-making work”)	“How do people work together in everyday settings to understand and plan the activities that need to be accomplished to put an intervention and its components into practice?”	The ways in which participants think about distinguishing use of digital systems from other formats for advance care planning, collectively agreeing on the purpose of EPaCCS^c,^ individually understanding what EPaCCS requires of them, and constructing potential value of EPaCCS for their work.
Cognitive participation (“relational work”)	“How do people work together to create networks of participation and communities of practice around interventions and their components?”	The ways in which participants become engaged in understanding what they need to do and support for EPaCCS to be sustained, influencing how EPaCCS use can be sustained, adapting to EPaCCS to support use by themselves and others, and supporting others’ engagement with EPaCCS.
Collective action (“operational work”)	“How do people work together to enact interventions and their components?”	The ways in which participants perform the tasks required for EPaCCS to support advance care planning, build accountability and maintain confidence in the use of EPaCCS, understand the appropriateness of existing roles and responsibilities relating to EPaCCS use, and view the resources and organizational support for EPaCCS use.
Reflexive monitoring (“appraisal work”)	“How do people work together to appraise interventions and their components?”	The ways in which participants appraise the effects of EPaCCS, themselves and with colleagues understand whether EPaCCS are operating well, individually understand and respond to the impact of EPaCCS, and modify their work in response to their appraisal of EPaCCS.

^a^Definitions derived from May et al (2022) [46].

^b^How definitions of NPT constructs were “in the simplest possible terms” [48] and applied to data collection, analysis, and interpretation.

^c^EPaCCS: Electronic Palliative Care Coordination Systems.

### Recruitment and Settings

Recruitment took place in 2 UK regions in London (population circa 9 million) and West Yorkshire (population circa 2.3 million). Participants comprised a subsample of respondents to an earlier survey who had agreed to be contacted for follow-up interviews. In West Yorkshire, EPaCCS comprise a template that is embedded within a patient’s electronic record, generally in the electronic record system used by primary care providers that can share information across different care settings within a defined geographical area. Within London, at the time of this study, the most used EPaCCS system was “Coordinate My Care” (CMC). This was a standalone system (eg, it operated outside routinely used patient records), and enabled patients to access their own records [14]. Since the conception of this study, it has been superseded by an EPaCCS system called “Universal Care Plan.”

Participants were initially selected using purposive maximum variation sampling to gather the widest range of perspectives [49]. This entailed sampling participants based on specific criteria (geographical location, professional role, setting of care, and levels of understanding of or engagement with EPaCCS based on previous survey responses). The logic underpinning this approach was to explore our research aim from diverse perspectives [49]. We worked across primary, secondary, and tertiary care settings in purposefully recruiting doctors, nurses, care home staff, paramedics, and general practitioners. Difficulties in recruitment, however, meant that we supplemented our recruitment approach with convenience sampling using the same criteria.

Participants were approached via email and provided with a participant information sheet. A combination of verbal and written informed consent was obtained, either by AB or JB, prior to interviews being conducted. Recruitment ran concurrently with data collection between November 2021 and June 2022. The concept of “information power” [50] was used to guide decisions on when to halt recruitment and data collection. This entailed several meetings during which research team members (AB, JB, MT, MA, CE, and KS) considered whether and when data collected from our sample held enough relevant and detailed information to comprehensively understand our research aim.

### Data Collection

Single, web-based interviews were conducted by 1 of 2 researchers, both of whom had prior experience in qualitative interviewing [AB (male, research fellow) and JB (female, research fellow)]. The topic guide (provided in Multimedia Appendix 2) comprised questions about how participants used EPaCCS, alongside the factors that they perceived affected their implementation. During development, these questions were mapped onto the 4 primary constructs of NPT. Interviews were audio recorded, anonymized, and transcribed verbatim. All participant personal data were stored in a secure cloud storage platform within password-protected files. These data were only accessible to, shared between, and used by members of the research team, using data-sharing agreements.

### Analysis

Interview data were managed using NVivo (version 12; Lumivero) [51] and analyzed using thematic Framework analysis [52]. The 4 primary constructs of NPT were used as the theoretical framework to guide the coding and interpretation of data. Data analysis included moving between induction and deduction. We first used NPT to deductively build our initial analytic framework and then supplemented this through inductive coding in which we explored how patterns grounded in the data related to and enriched our analytic framework. Analysis consisted of seven iterative steps: (1) familiarization (through rereading transcripts), (2) coding (by labeling relevant segments of transcripts that aligned with our research aims), (3) creation of an initial analytic framework (by grouping similar codes into categories and categories into themes), (4) indexing (by applying our analytic framework back to raw data and refining it where appropriate), (5) charting (by creating a matrix that explored differences in data across region, role, and setting of care), (6) description (through defining and describing themes), and (7) interpretation (using our theoretical framework to further interpret our findings through the write-up of data). This approach allowed us to conduct within- and between-case pattern matching to explore where participant accounts on the use and implementation of EPaCCS converged or diverged, and how this was influenced by contextual factors (ie, setting, region, and role). Data analysis was led by AB, with fortnightly meetings between authors (AB, JB, MT, and MJA) to review the ongoing coding and analysis.

Multiple approaches were used to ensure rigor during data analysis. Throughout data collection and analysis, the researchers engaged in different forms of reflexivity. This included reflecting introspectively (inward reflections on how they impacted the research process and vice versa) and intersubjectively (reflections on relationships between them and participants) [53]. These were used as a “springboard for interpretations and more general insight” into the ways through which understandings of the research aim were being co-constructed through the research process, including analysis [53]. This included regular discussions with JB, who shared data collection and who was familiar with the data corpus. Moreover, throughout the analytical process, members of the wider interdisciplinary research team (consisting of academics and clinicians with experience in palliative care research and practice from across care settings including hospital, community, and primary care) acted as “critical friends” [54]. This entailed working collaboratively through regular meetings and written feedback in which findings were constructively challenged, reflexivity encouraged, and alternative interpretations of the data proposed. This process took place until the research team agreed that the final analytic framework accurately reflected participant accounts.

### Ethical Considerations

The North of Scotland Research Ethics Committee approved the research (reference 21/PR/0428). In this study, we also recognized ethics as a reflexive process through engaging in “ethics in practice” [55]. This approach was used to remain responsive to and navigate ongoing and potentially unexpected ethical issues that may have arisen throughout data collection, analysis, and write-up (eg, by reflecting on how the research might affect professionals’ clinical practice and potential impact on patients and carers). All participants provided written consent prior to participating in the study. Before analysis, all interview transcripts were deidentified and stored on a secure cloud storage platform only accessible to the study team. Organizations in which participants were based were offered reimbursement of £75 (US $95.91) for allowing a professional to participate in the study. The level of the incentive payment was based on the cost of 1 hour of a locum doctor in the United Kingdom and was agreed upon by the research ethics committee and study funder.

## Results

A total of 52 people (characterized in Table 2) participated out of the 99 people approached for interview participated (characterized in Table 2), from London (n=29) and West Yorkshire (n=23). These participants represented a range of different professionals who work across hospice, primary care, care home, hospital, and community settings.

**Figure 1:**
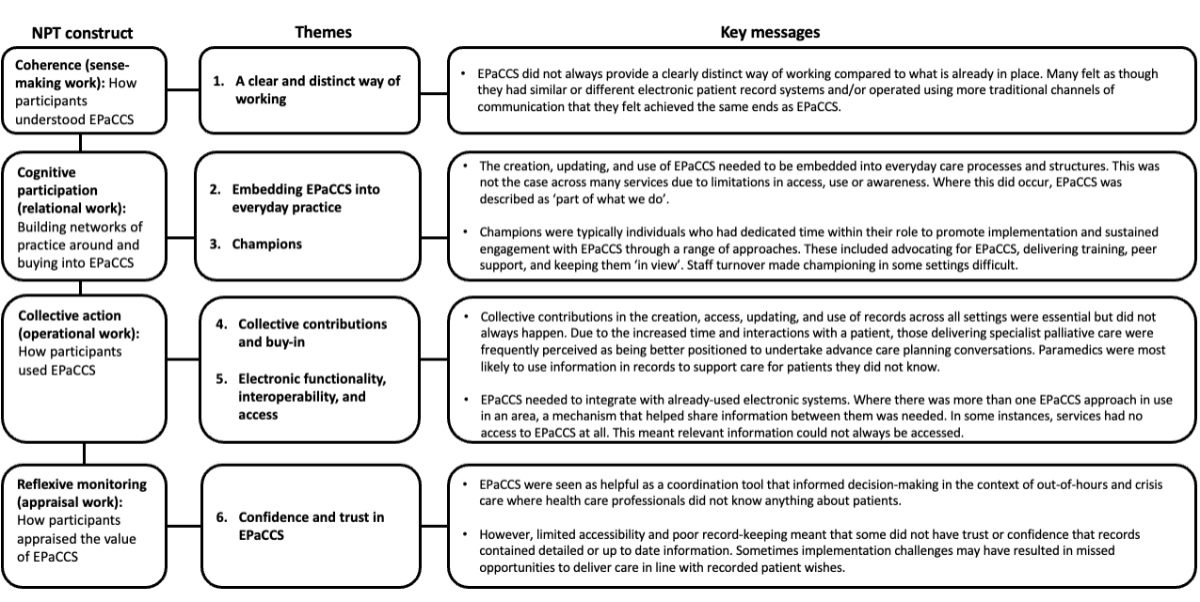
An overview of the themes and key messages generated from the thematic framework analysis as they align with the 4 constructs of normalization process theory. EPaCCS: Electronic Palliative Care Coordination Systems.

Six themes were developed and are represented under the corresponding constructs of NPT (coherence, cognitive participation, collective action, and reflexive monitoring). Figure 1 provides an overview of these main themes.

**Table 2:** Characteristics of study participants (N=52) including location, settings of care, and role.

		London	West Yorkshire	Total
**Interviews, n**		29	23	52
**Setting of care, n**				
	Hospice	8	4	12
	Primary care	7	5	12
	Care home	5	4	9
	Hospital	5	3	8
	Ambulance	3	3	6
	Community nurse	1	4	5
**Role, n**				
	Registered nurse	6	7	13
	Care home	4	3	6
	Community	1	4	5
	Hospital	1	1	2
	General practitioner	7	5	12
	Palliative care consultant	6	4	13
	Paramedic	3	3	6
	Clinical Nurse Specialist	4	1	5
	Care home manager	1	2	3

### Coherence: How Participants Understood EPaCCS—Theme 1: A Clear and Distinct Way of Working

For a complex intervention to be normalized, it needs to be understood as a clear and distinct way of working that enhances patient care. In the case of EPaCCS, this required health care professionals to appreciate how adding all relevant advance care planning information onto a single digital platform allowed for easier sharing among organizations. Across all settings of care, however, participants did not always see how EPaCCS enhanced patient care and outcomes compared to traditional channels of communication. These included using paper-based discharge summaries, cover letters, face-to-face conversations, emails, and telephone for direct communication of advance care plans across settings:

I don’t think it [EPaCCS] changes much in terms of our GP engagement with patients … our primary channel of communication is telephone, face to face, perhaps email and EPaCCS doesn’t sort of sit with any of those. Our primary function really is to provide the clinical care and record that clinical care and those are traditional methods of doing that. EPaCCS is an add-on, and I think because it’s an add-on, it’s not the primary form of communication.

Lon18, GP, London

Participants working in care homes across London and West Yorkshire reported using a range of different electronic patient record systems for documenting their residents’ care plans. These systems facilitated the documentation of patient wishes and preferences that could be easily accessed, reviewed, and updated internally by care home staff. Some electronic record systems used in care homes were also capable of integrating and sharing data with other systems across different settings, but this was often limited to those in general practice and not on the scale envisaged by EPaCCS:

The electronic care plan system that we are using, it’s something called PCS, so Person Centred Software. You can do your usual stuff like you said, day-to-day care notes and things like that. There also with PCS, the party piece it has is something called GP connect … with PCS if you do kind of speak with them, they are able to kind of merge certain systems together with their [other healthcare settings’] system … as far as I know PCS works with all of them

WY12&WY13, care home managers, West Yorkshire

### Cognitive Participation: How Participants Built Networks of Practice Around and Bought Into EPaCCS

#### Theme 2: Embedding EPaCCS Into Everyday Practice

The service-wide embedding of EPaCCS into everyday practice varied and was partly influenced by the extent to which they were an integral part of everyday care processes, structures, and settings. In some contexts, entire services had no access to EPaCCS. Where EPaCCS were present and accessible across multiple settings, implementation was still thwarted because they were not used consistently by some health care professionals:

it can be like an easy thing to do and I think it gets ingrained in your normal kind of process of doing a patient’s notes … but I think when people just don’t know about it or don’t know how to access it, it then becomes, it seems more of a challenge to actually set one up whereas once you’ve got used to it, it just becomes part of your normal routine first assessment, set up CMC and then it’s done.

Lon5, hospice community doctor, London

Some participants reported that EPaCCS were integrated into everyday clinical routines. In these instances, EPaCCS were referred to as being “part of what we do” [WY2, community/hospice, West Yorkshire]. This included ensuring that EPaCCS management was integrated into key care processes such as initial assessments, caseload reviews, admission and discharge planning, multidisciplinary team meetings, handover sheets, and standard operating procedures:

Whenever we take on a new patient onto the caseload, we will do our level best to have a conversation about the current EPaCCS system we use, which is Coordinate My Care, to get consent to put people on that system … it’s very much part of our mantra, it’s something that we do … We talk about Coordinate My Care at our weekly multidisciplinary team meetings, so we ask people to check that people are on there and if they’re not, we try and think of a plan to get somebody on there.

Lon26, hospice consultant nurse, London

#### Theme 3: Championing Driving Implementation

Participants across all professional groups in both regions reflected on the role of colleagues who championed the use of EPaCCS. Champions were typically individuals who had been given dedicated time within their role to promote implementation and who encouraged staff engagement with EPaCCS. Champions used a range of approaches including offering peer support, taking active roles in teaching and education, presenting the potential benefits of EPaCCS, and keeping them present or “in view” in everyday clinical practice:

Having local champions who are just, [I] don’t want to say checking, but just ensuring that locally they’re being completed, that they’re demonstrating a difference. You know, there’s no point doing it if it’s not demonstrating any benefit really is there?

WY2, community/hospice consultant, West Yorkshire

There were examples where engaging staff in the use of EPaCCS and learning how different systems work was described as a “constant recurring battle” [Lon06, hospital Clinical Nurse Specialist, London] that required time, dedication, and energy. This view was particularly present in hospital settings and the challenge was mainly attributed to high staff turnover. Different staff meant that the same messages and training had to be repeated continuously for EPaCCS to remain a priority for teams. However, such training did not always translate into increases in health professional use:

The reality is, I think that EPaCCS is underused, but generally across the hospital. That's why I have to be out there doing education and encouragement… it's continually education, trial, training, nudging, pushing to get them to use it because, a level of busyness, a churn of staff, you know they’re churning staff all the time, they’re coming from different Trusts who are not used to SystmOne, never mind EPaCCS, so it's a continual, continual, continual thing and training

WY7, hospital nurse, West Yorkshire

### Collective Action: How People Enact EPaCCS

#### Theme 4: Collective Contributions and Buy-in

Collective contributions referred to the extent to which health care professionals across settings of care contributed to the creation, sharing, updating, and use of EPaCCS records to inform care. Underpinning collective contributions was the degree to which health care professionals “bought into” EPaCCS by seeing them as a legitimate part of their role or as supporting the work of others. There was a general agreement that EPaCCS needed “buy-in *from everyone - not just palliative care teams – for it to work*” [WY2, consultant, community/hospice consultant, West Yorkshire]. Despite this, health care professionals working in specialist palliative care were often the ones creating and updating EPaCCS records.

Participants had different perceptions in terms of the skills and capacity of professionals across different care settings to support advance care planning. Some felt that staff working in specialist palliative care were best placed to initiate sensitive conversations about advance care planning and end-of-life choices. Others reflected that although they believed health care professionals working outside of palliative care settings could broach advance care planning conversations, they did not always have the confidence to do so. Indeed, participant accounts suggested that when palliative care services were not involved in a patient’s care, this led to a general lack of clarity over who should do what, when, and how, which often resulted in the ad hoc creation of records.

If they’re working in specialist palliative care, most of those people will have those skills [for advance care planning]. If we then look at people who don’t deal exclusively with palliative care but see a lot of it, so district nurses, elderly medicine doctors, general practitioners, I think there is a lot of skill there. There’s not always the skill and there’s often a gap in confidence to apply the skills that people have … when people don’t have the skills and confidence, that first conversation where we seek, where we explain to the patient where they are in their illness and the fact that they’re in a palliative phase of their illness and seeking consent to use an EPaCCS doesn’t happen

WY6, hospice, West Yorkshire

…in [our] Community Trust we’ve also got a respiratory service who are involved with people that are end stage of respiratory failure and we also have heart failure nurses and diabetic nurse specialists, and you know, those sorts of questions are talked about with patients often. But what we’re trying to do as a service in palliative care [non-specialist palliative care in Community Nurse Team] is encourage that to be done because it isn’t done as much as it should be really. You know when people are actually reaching sort of end-stage heart failure but yet nobody’s actually spoken to them about their wishes at the end of life. They feel it’s not their responsibility.

WY9, community nurse, West Yorkshire

A concern among participants working in general practice was that it was difficult to contribute to EPaCCS because they did not always fit with their existing ways of working. Given the time constraints and competing priorities in general practice, accessing an EPaCCS record and then conducting and documenting advance care planning conversations was often seen as unmanageable and unrealistic:

we’ve only got a 10- or 15-minute window to see that patient for their current problem, so we don’t bother to update the CMC after just because of sheer time. So, unless you’re having a special CMC kind of session and you’ve dedicated a bit of time to go and do a home visit on someone or you’ve planned it in that you’re going to update the CMC and those wishes and concerns etc, that’s only when it really gets touched by the GP practice.

Lon16, GP, London

Conversely, paramedics with access in London were likely to use EPaCCS records, typically because it helped decision-making around the urgent management of patients that they were hitherto unfamiliar with:

…we use it just as part of our decision making … I would say it’s a big part of my role… in the main bulk of my role which is in an ambulance setting I use it all the time, it’s second nature and it’s very valuable.

Lon29, paramedic, London

#### Theme 5: Electronic Functionality, Interoperability, and Access

At a technical level, the integration of EPaCCS within existing electronic systems was important to their implementation. However, according to participants, this process of integration had not always occurred. Across most care settings in London, participants were frustrated that CMC was not seamlessly interoperable with existing electronic patient record systems. This lack of technical interoperability (ie, basic data exchange capabilities between systems) created a restrictive process that resulted in additional work because health care professionals had to remember (and frequently update) log-in details and enter duplicate information across different systems:

Coordinate My Care for us is a completely separate system … it doesn’t pull data from the current electronic system, you still have to kind of manually enter the patient’s name, address, NHS number … it is extra work … that kind of influence[s] how detailed a care record might be. Sometimes we will just put on the basic information that you think’s important … if it was integrated into a current kind of electronic system then I guess it would just make it easier.

Lon5, hospice community doctor, London

In services across West Yorkshire, problems were caused by the fact that EPaCCS were embedded within several different electronic health record systems. However, because no mechanism in place allowed for the sharing of information between these systems, health care professionals could not always access relevant information from EPaCCS records when they needed it:

In this area a lot of folks were using SystmOne and we use EMIS … So, they [hospice services] can’t see what we’ve done on our system and it’s a bit messy … I know EPaCCS is supposed to be a document that everyone can access and fill in, but you can’t, ours is just on our system and ‘cos no one else can fill it in or see what’s been changed that’s where it falls down…. it is stupid that you can’t share an EPaCCS with anyone, it seems like a bit pointless

WY17, GP, West Yorkshire

### Reflexive Monitoring (“Appraisal Work”): Appraising the Value of EPaCCS—Theme 6: Confidence and Trust in EPaCCS

Participants across different regions and settings reflected on the potential value of EPaCCS as a tool that could facilitate the coordination of care. Others reflected on first-hand experiences of how EPaCCS were valuable in the context of crisis and out-of-hours care. In particular, paramedics who had access felt that EPaCCS provided vital information needed to effectively support person-centered decision-making with people whom they did not know:

If I saw a CMC for example where it was recorded that the patient had a preference for treatment care in the home, that would make it much more likely that I would dispatch one of my colleagues because we already know that that is the patient’s preferences and so as far as possible, we’re going to work to make that happen … End of life care pushes against the normal direction of paramedic care, [the] normal direction of paramedic care is rescue, save … end of life care obviously isn’t about life-saving, it’s about dignified death, symptom control management. It’s a change of thinking and so a lot of paramedics struggle with that, and I think they will look for anything that will help them guide them in that process and I think CMC is one that people are very familiar with using and generally find quite helpful.

Lon29, paramedic, London

I’ve worked with the ambulance service in the time before it [EPaCCS] was standard practice and I would say it’s such a necessity now that we’ve got it, if we lost it I think it would literally be like losing my hand … it cuts out awkward conversations and it also cuts out us doing something that may be an issue would be against their wishes … it takes that anxiety or the uncertainty out of the what are we going to do and see what’s best for this patient.

Lon28, paramedic, London

However, other professionals reported that EPaCCS records were sometimes of poor quality and that there were frequent problems with accessing the system. Consequently, this reduced their confidence that EPaCCS records contained sufficiently up-to-date information to support decision-making. Concerns were not about the potential of EPaCCS to improve care but regarding implementation issues such as restricted access, shareability, and inconsistent use of these records by staff. There was a fear that such problems could lead to patients receiving interventions and treatments that were against their stated and recorded wishes and preferences. This was particularly the case for paramedics who, without access to records, were more likely to make risk-averse decisions to hospitalize patients in the absence of knowing their wishes:

I can tell you categorically that we have not acted in the patient’s best interest … I’ll have taken somebody into hospital without my knowledge that [they have] an end-of-life care plan somewhere

WY21, paramedic, West Yorkshire

## Discussion

### Principal Results

This study explored factors that influenced the implementation of EPaCCS in routine clinical practice across different care settings in 2 major regions of England. It identifies and elaborates on challenges around the implementation of EPaCCS, including problems with access, and inconsistent use and engagement across settings. A key issue was technological limitations, with separate electronic health records often operating in parallel systems or failing to provide sufficient documentation or access. Such problems have led to the potential value of EPaCCS being unrealized.

**Figure 2:**
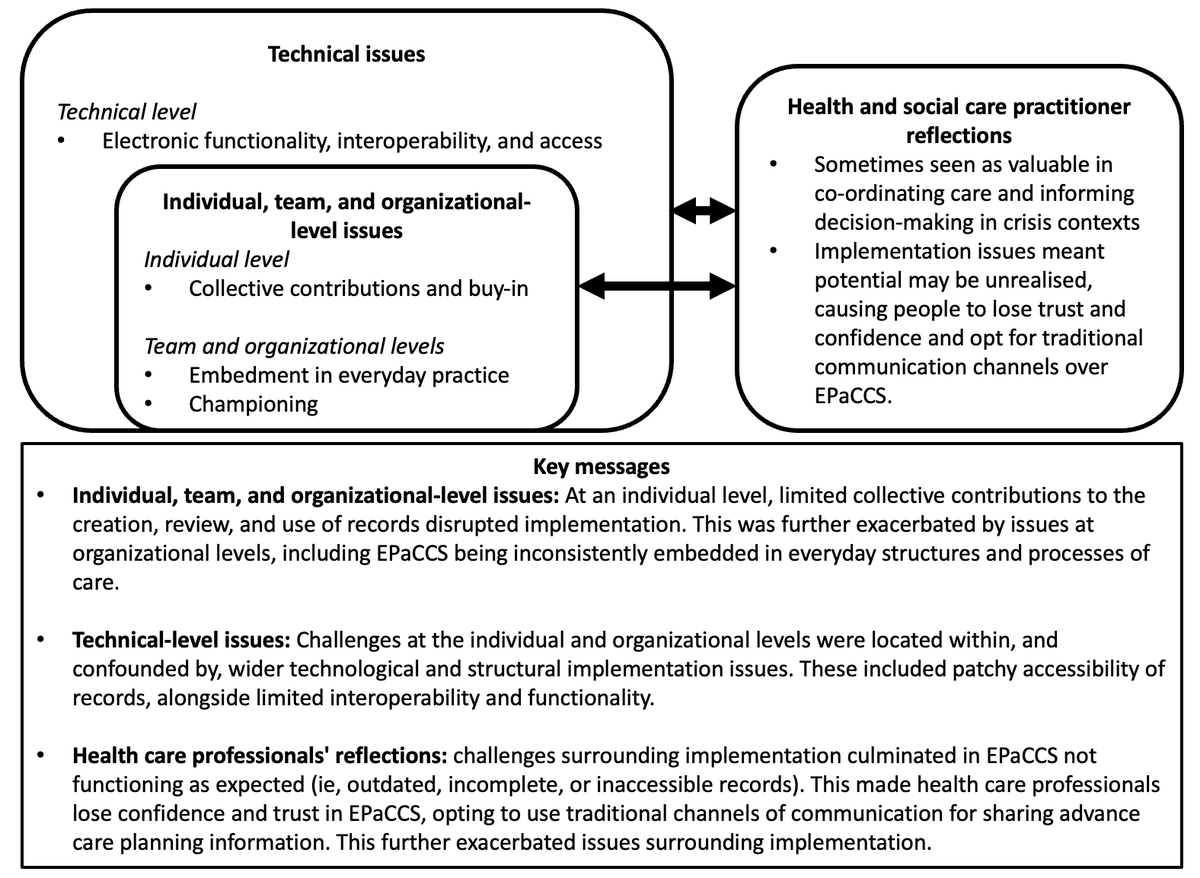
An overview of the individual, team, organizational, and technical factors that affect the implementation of digital advance care planning systems (ie, EPaCCS) in routine practice. EPaCCS: Electronic Palliative Care Coordination Systems.

Guided by sociotechnical systems theory [56], Figure 2 summarizes these issues by highlighting how interactions between the individual, team, organizational, and technical dimensions of EPaCCS affected implementation. The content of this figure is grounded in the data. It was generated inductively through highlighting relationships between each of our themes alongside how they related to different levels of practice. This allowed us to move beyond description by explaining linkages between themes and bringing them together in a way that tells an overarching story of health care professionals’ perspectives on the processes that influenced the implementation of EPaCCS.

### Comparison With Prior Work

A key theme in this study was that limited buy-in and collective contributions to the creation, initiation, and use of EPaCCS across care settings affected implementation. Previous research has primarily used quantitative approaches to explore EPaCCS implementation, such as determining the proportion of EPaCCS records created across a specified population, and the average number of days that EPaCCS records are created before death [27,34]. However, our findings add novel and unique contributions by describing who initiates EPaCCS records, the factors that influence this, and the differences across regions. While some general practitioners and community nurses reported recording advance care planning discussions electronically, our study also highlights contributions made by health care professionals working across specialist palliative care settings. For some participants, providers of specialist palliative care were perceived as being better placed to initiate advance care planning conversations. A referral of a patient to specialist palliative care was perceived as affording greater time to interact with patients and other staff groups, alongside their experience in facilitating advance care planning discussions with patients and their families.

Participant experiences resonate with previously documented challenges related to advance care planning such as perceived lack of time, hesitancy in initiating conversations, and lack of care continuity [57-59]. Lack of clarity over who contributed to records and the timing of these contributions often resulted in poor-quality data. Such issues led to fears that EPaCCS records were incomplete or out of date, with some records being overly detailed or conversely, insufficiently informative to effectively support decision-making in out-of-hours or emergency care. The successful implementation of interventions that work across organizations (such as EPaCCS) relies on “whole systems” thinking [35]. Such implementation entails those involved in the use and implementation of EPaCCS (including commissioners and those responsible for service [re]design) accounting for the needs and influence of people working across different care settings and specialties.

In this study, care home staff reported having detailed discussions regarding residents’ wishes and preferences for care and documenting these within their own electronic systems. This aligns with previous research that the close relationships between care home staff and residents mean that staff are also well placed to have the sensitive and in-depth conversations required for advance care planning [60]. In this study, while electronic systems were reported to be used and engaged across care homes, the information contained within them was mostly inaccessible to external services. Care homes were also largely unable to access or provide helpful and detailed information from EPaCCS that could be used by other services. This highlights a key gap in the “whole systems” approach to EPaCCS. Addressing this gap is especially important given that care homes are projected to be the most common place of death in England by 2040 [61].

The implementation of EPaCCS at the individual, team, and organizational levels was affected by wider technological challenges. Issues related to the technical dimensions of EPaCCS have been well documented in the literature [13,30,33,36]. However, this study provides novel findings relating to health professional experiences of EPaCCS, further strengthened by its collection of data across 2 large geographical regions. This study builds on previous work and contributes details of how and why issues with access, functionality, and technical interoperability (ie, data integration, presentation, and exchange) [62] affected the implementation of EPaCCS across the United Kingdom. In London, challenges were experienced around the ability to move data between EPaCCS and existing electronic patient record systems (and vice versa) without duplication. Across West Yorkshire, a major interoperability issue was the failure of different electronic patient record systems to exchange advance care planning information. Moreover, while paramedics were most likely to use information within EPaCCS records in London to support decision-making in crisis contexts, they did not have this access across West Yorkshire. While these interoperability limitations affected health care professionals in different ways, they ultimately hindered the harnessing of the electronic information sharing and care coordination that EPaCCS is intended for.

### Implications for Policy and Practice

Our findings highlight implications for practice relating to the use and implementation of electronic systems for palliative and end-of-life care. For complex interventions like EPaCCS to become normalized into everyday practice, they must fit within and enhance established systems of care [63]. It is also crucial that users can see their benefits to patient care and clinical practice compared to traditional ways of working [64,65]. However, these findings suggest that, in their current format, EPaCCS are not working as intended for facilitating person-centered care. Implementation challenges which resulted in missed opportunities to deliver care in line with recorded patient wishes, sometimes led to a loss of trust and confidence in EPaCCS, instead staff opted for more traditional means of communication and a return to traditional communication methods. Consequently, a redesign of EPaCCS is likely to be necessary to achieve the optimal configuration for successful implementation across different settings of care and geographical locations. To this end, we have generated a set of questions focusing on factors that were found to be influencing the uptake and use of EPaCCS across different settings of care (see Table 3).

**Table 3:** Questions for health care practitioners, commissioners of health care services, and policy makers to consider in optimizing the uptake and implementation of electronic information-sharing systems across different settings of care.

Level of action	Relevant to	Questions to consider
Individual or user	Professionals across settings of care involved in the creation, updating, reviewing, and use of electronic information sharing systems to inform decision-making	What are current levels of staff confidence in broaching and conducting advance care planning conversations with patients who have life-limiting illnesses? To what extent are professionals across care settings clear on whose responsibility (including their own) it is to engage in advance care planning and documentation of any resultant patient wishes and preferences? What are current levels of staff awareness of the different stages of electronic information sharing (eg, the creation, update, review, and use to inform decision-making) relating to their role?
Team or organizational	Team leaders and service management	Is there collective agreement across care teams on how to embed electronic information sharing systems within everyday structures and processes (eg, at admission or discharge, multidisciplinary team meetings, and handovers)? Is there a dedicated member or group of staff that can help to monitor uptake and promote sustained engagement with electronic information sharing systems both within and between services? Is there clarity on the way in which electronic systems are intended to be used for the documentation of advance care plans (eg, which patients it is intended for, and when it might be used as part of their care)?
Technical or structural	Commissioners and policy makers	Do all services that are involved in the care of people with life-limiting illnesses have access to an electronic information sharing system (including paramedics and care homes)? Have the outcomes of the electronic information-sharing systems been agreed upon, alongside how outcomes can be measured and fed back to users?

While this study was conducted within England and discussed within the policy context of the United Kingdom, the policy implications have international relevance. The global strategy on digital health from the World Health Organization calls for the implementation of functional and interoperable electronic health records that can contribute to informed decision-making and high-quality, person-centered care [66]. Our findings highlight factors to consider when developing electronic systems for use in the delivery of palliative and end-of-life care. The questions presented in Table 3, therefore, are likely to have relevance to policy makers and practitioners seeking to use and implement similar complex digital interventions (including electronic information-sharing systems) across multiple country and health care contexts. With increasing governmental policies on the development and implementation of health information technologies within the United Kingdom [62], these questions can guide efforts in the context of palliative and end-of-life care.

### Strengths and Limitations

A strength and novelty of this work lies in the adoption of NPT to explain the processes across different levels of the health care system which affected whether and how EPaCCS were normalized into everyday practice. Through recruiting a wide range of health care professionals, across 5 settings of care, and 2 major regions within the United Kingdom, naturalistic generalizations [67] may be made from this work. That is, the findings of this study are likely to resonate with the personal experiences of healthcare professionals who use EPaCCS and similar health information technologies across end-of-life settings. We highlight 4 study limitations. First, we struggled to recruit community nurses across London, meaning that the application of study findings to this professional group is likely to be limited. Second, this study only sought the perspectives of health care professionals. Future research should explore patients' preferences on the content, sharing, and accessibility of their electronic records, alongside the impact of such digital interventions on the patient experience and clinical outcomes. Third, in line with other studies [68] throughout analysis, we found that the technical language of NPT and the overlapping of its components made deductively coding and interpreting data using this theoretical framework challenging. Fourth, we also appreciate that constructs comprising NPT focus on specific factors that influenced the implementation of EPaCCS in routine practice. Other implementation theories, models, and frameworks (eg, the Consolidated Framework for Implementation Research [69], Promoting Action on Research Implementation in Health Services [70], Capability, Opportunity, Motivation and Behaviour Theory [71]) may have provided different, yet equally valuable insights into answering the research question.

### Conclusions

The successful implementation of digital interventions into routine care depends on the extent to which they enhance established ways of working with minimal disruption. EPaCCS represents just 1 approach to the electronic sharing of advance care plans, and other forms of digital advance care planning exist internationally. Integral to the implementation of digital advance care planning systems for palliative care is ensuring they can allow health care practitioners to document, access, use, and share information across multiple care settings. There also needs to be an effort at individual, team, and organizational levels to make sure that these tools are embedded into everyday care practices. It is paramount that they are championed within and between services, and that people know when, how, and why to use them. Commissioners, health care services, and practitioners should consider these multilevel factors when planning and rolling out digital advance care planning approaches.

## Appendix

Multimedia Appendix 1 COREQ (consolidated criteria for reporting qualitative research) checklist.

Multimedia Appendix 2 Interview topic guide.

## Abbreviations

CMC: Coordinate My Care
COREQ: consolidated criteria for reporting qualitative research
EPaCCS: Electronic Palliative Care Coordination Systems
NPT: Normalization process theory

## Data Availability

Deidentified transcripts generated and analyzed during this study are available from the corresponding author on reasonable request.
