# Supplementary material for: Factors Influencing the Implementation of Digital Advance Care Planning: Qualitative Interview Study
Source: J Med Internet Res. 2024 Aug 16;26:e50217. doi: 10.2196/50217 (PMC11364948; doi:10.2196/50217)
Supplement: Multimedia Appendix 2 [file jmir_v26i1e50217_app2.docx]

| 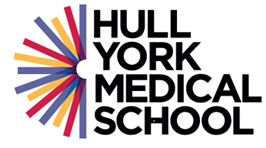 | 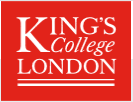 | 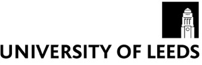 |
| --- | --- | --- |

**OPTIMAL CARE: Topic Guide for interviews with health and social care professionals**

*Please note the interview topic guide is a living document. It is not the intention to ask ALL of the questions. The design is iterative to tailor the interview to the needs of the participant, and the issues that they feel are most important. As analysis occurs concurrently the topic guide is likely to be adapted over time - questions might be added, amended or omitted – and used flexibly.*

*Additional questions related to the main issues arising from the WP2 Survey will be inserted as appropriate to the participant and their role.*

**Study title:** OPTIMAL CARE (Optimising Palliative Care through electronic coordination)

1. **Introduction**

- Thank you for agreeing to take part in this interview.
- As you know, we are talking to individuals working in teams that see patients who are receiving palliative care. We are talking about their understandings, views and experiences of using EPaCCS in end of life care.
- With your permission I would like to record the interview; all details will be confidential.
- Do you have any questions or concerns?

1. **Consent**

- Review the information sheet and opportunity to ask questions
- Obtain written consent
- Complete demographic information

**(Start audio-recorder)**

**Interview questions**

1. **About you (very brief)**

Please would you tell me about your role in end of life care?

- - Patient facing, managerial, type of service, etc
  - Do you have any responsibility for overseeing any activity relating to electronic patient records

What do you think about the value of using EPaCCs as part of your routine practice?

- - Why do you think that?

1. **Using the EPaCCS to document and share (in depth; mapped to the different components of NPT)**

**Collective action (Operationl work; the ways in which people – individually and collectively – work to together in using and implementing EPaCCs)**

Could you talk me through the process of when and how you use EPaCCs in your everyday practice?

| **General probes** | |
| --- | --- |
| - - What electronic patient record systems do you use?   - What info is recorded on these systems? | |
| **Probes for those who create/initate EPaCCs** | **Probes for who do not create EPaCCs (but are on receiving end of them)** |
| - How you initiate, review and update records for patients in your service? - How you access, review and update information about patients that are initiated in other services? - How does it affect how you engage with patients? (e.g., do they support continuity of care and the communication of advance care planning decisions/preferences)? - How are they communicated with other services and healthcare professionals? | - How you access and review information about patients that are initiated in other services? - How are they communicated with other services and healthcare professionals? - How does it affect how you engage with patients? (e.g., do they support continuity of care and the communication of advance care planning decisions/preferences)? |

How do you find this process?

- Do you feel as though you’ve experienced any challenges when using EPaCCs? (e.g., usability/resources/time/confidentiality/access/technical/patient acceptability)

What do you think needs to be in place to address these challenges?

Do you feel as though you and those you work with have the skill sets and confidence needed for EPaCCs to be effective?

- Why?
- What would need to change?
- Are there any specific training needs that need to be addressed?

Are there any unintended consequences of using EPaCCs that you have experienced or could forsee?

**Cognitive participation (Relational work: What people do to engage in EPaCCs in order to legitimise, and build a community of practice around, their use)**

Are EPaCCs something you see as part of your role?

Does the team and setting that you work in influence your use of or interaction with EPaCCs?

- Do team members see the point of EPaCCs?
- Are there any beliefs or attitudes among your team or across settings that has supported your use of, (or engagement with) EPaCCs?
- How?

Is there somebody who drives the use of EPaCCs in your service?

- What is their impact on implementing EPaCCs? OR What is the impact of not having this?

How confident are you that other healthcare professionals can access electronic plans you have initiated in a timely way?

**Coherence (sense-making work: How individuals and groups understand what EPaCCs are and how/when to use them)**

What do EPaCCs mean to you in relation to your role?

Do you think that there is a shared understanding of what EPaCCs are within the organisation that you work?

- - What is this?
  - Is there a shared understanding of EPaCCs with other organisations and healthcare professionals that you work with?
  - How do these shared understandings impact the way you work with EPaCCs?

What do you think the intended outcomes (benefits) of using EPaCCs are?

Are EPaCCs easy to describe to other people (e.g., patients and other professionals)?.

- How do you think patients/families/other professionals understand EPaCCs?

**Reflexive monitoring (Appraisal work: how people appraise and assess the value of EPaCCs after using them)**

If any, what do you think the benefits to using EPaCCs are and to whom?

- Patients?
- Families?
- Other healthcare professionals?
- … why?

Are these benefits valued by families and others you work with? (could also explore if families seem to see the benefit or if it is something they just agree to)

Do you think that any changes need to be made in order to make EPaCCs work better?

- What would these be?
- Why?

What do you feel would be needed to sustain the implementation and use of EPaCCs in the long term in your workplace?

- Individual, team, and organisational factors?

What information do you think is important to capture in order to measure the whether EPaCCs are useful or not?

1. **Feedback (5 minutes)**

Probably just – is there anything else about use of EPaCCS?

Have these questions allowed you to talk about the most important issues for you?

Is there anything else you think would be useful for us to know?

Finally - Introduce Work Package 5 - if the participant is interested we are sending invitations and information to everyone who expresses an interest - however places will be limited so they will be asked to book as soon as they make their decision

**Thank you for your time.**
